# Interference-Free HER2 ECD as a Serum Biomarker in Breast Cancer

**DOI:** 10.4172/2155-9929.1000151

**Published:** 2014-11-14

**Authors:** Lian Lam, Brian J. Czerniecki, Elizabeth Fitzpatrick, Shuwen Xu, Lynn Schuchter, Xiaowei Xu, Hongtao Zhang

**Affiliations:** 1 Department of Pathology and Laboratory Medicine, University of Pennsylvania, Philadelphia, U.S.A; 2 Department of Surgery, University of Pennsylvania, Philadelphia, Pennsylvania, U.S.A; 3 Division of Hematology-Oncology, HUP, 16 Penn Tower, Philadelphia, U.S.A

**Keywords:** HER2/neu, HAMA, HAIA, Survival, DCIS, Vaccination

## Abstract

Over-expression of the HER2/neu receptor occurs in 20 to 30 percent of breast tumors and is linked to poorer prognosis. The HER2/neu expression status determines whether or not patient will receive trastuzumab-based treatment. In clinical practice, over-expression of HER2/neu is routinely identified using Immunohistochemistry (IHC) or Fluorescence in Situ Hybridization (FISH), both of which are invasive approaches requiring tissue samples. Serum assays for the Extra Cellular Domain of HER2/neu receptor (HER2 ECD) have been reported but the use is very limited due to serum interference factors (e.g. human anti-animal immunoglobulin antibodies) that lead to false test results and inconsistency with tissue Her2 status. We have developed an ELISA based approach using an MBB buffer to eliminate false results and to obtain more accurate assessment of HER2 ECD levels. Using this refined assay we retroactively measured HER2/neu levels from breast cancer patients and controls. Abnormal HER2 ECD levels were detected in about 32% of invasive breast cancer patients but not in controls or patients with benign diseases. In addition, we also showed that patients with elevated serum HER2 levels appeared to have worse survival regardless of treatments. In a small group of 12 Ductal Carcinoma in situ (DCIS) patients who received HER2/neu peptide vaccination and surgery, only one patient showed constantly rising HER2 levels after treatment and this patient had recurrence of HER2 positive tumor within 5 years. Our studies indicate that once the serum interference issue is resolved, serum HER2 ECD can have potential clinical utility to supplement the tissue based tests.

## Introduction

Over-expression of the human *her2/neu* gene, the homologue of the oncogene *neu*, is one of the predominant transformation-activating mechanism and achieves the same effect as the oncogenic mutations observed in the *neu* oncogene [1]. Indeed, the amplification of the *her2/neu* gene and over-expression of the related HER2/neu receptor are observed in 20–30% of primary human breast tumors and are correlated with poor prognosis and disease progression [2,3]. Specifically, an association between the extent of *her2/neu* amplification and the presence of tumor in lymph nodes was observed [2]. Furthermore, *her2* gene amplification was found to be a valuable predictive factor for overall survival and disease-free survival in individuals with tumors in their lymph nodes.

The extra cellular domain of HER2/neu (HER2 ECD) can be cleaved and released from the cell surface into circulation[4]. ADAM10 was identified as one of the critical metalloproteinases responsible for the cleavage of HER2/neu [5]. Shedding off the ECD leads to a truncated form of p95HER2 [6], which is implicated in the resistance to anti-HER2 antibody based targeted therapies [7]. Although alternatively spliced form of HER2/neu has been reported to encode the ECD (a.a. 1–633, termed p100) [8], some evidence argues against the splice variant as the main mechanism to produce HER2 ECD in the circulation: 1. Metalloproteinase inhibitors greatly reduced the HER2 ECD levels [5,6]; 2. Late stage breast cancer patients are more likely to have elevated HER2 ECD [9], while the p100 splice variant has been shown to functionally inhibit the proliferation of tumor cells [10].

Immuno-detection of serum HER2 ECD has been developed with various anti-HER2 antibodies [11]. Although FDA-cleared serum tests are commercially available, they are not broadly used in clinical practice. One problem associated with the serum test in sandwich ELISA (Enzyme-Linked Immunosorbent Assay) is the serum interference, which ismostly caused by the Human Anti-Animal Immunoglobulin Antibody (HAIA) or more commonly known as the Human Anti-Mouse Antibody (HAMA) [12]. To eliminate this problem, we have developed the MBB buffer [13], which was designed to prevent the weak interactions between capture/detection antibodies and HAIA but to spare the strong interaction with specific antigens. In this report we studied the HER2 ECD levels in breast cancer patients with the help of the MBB buffer. Our study indicated a potential clinical utility for the opimized serum HER2 assay to supplement the tissue tests and assist breast cancer treatments.

## Materials and Methods

### Patients

**Figure 1:**
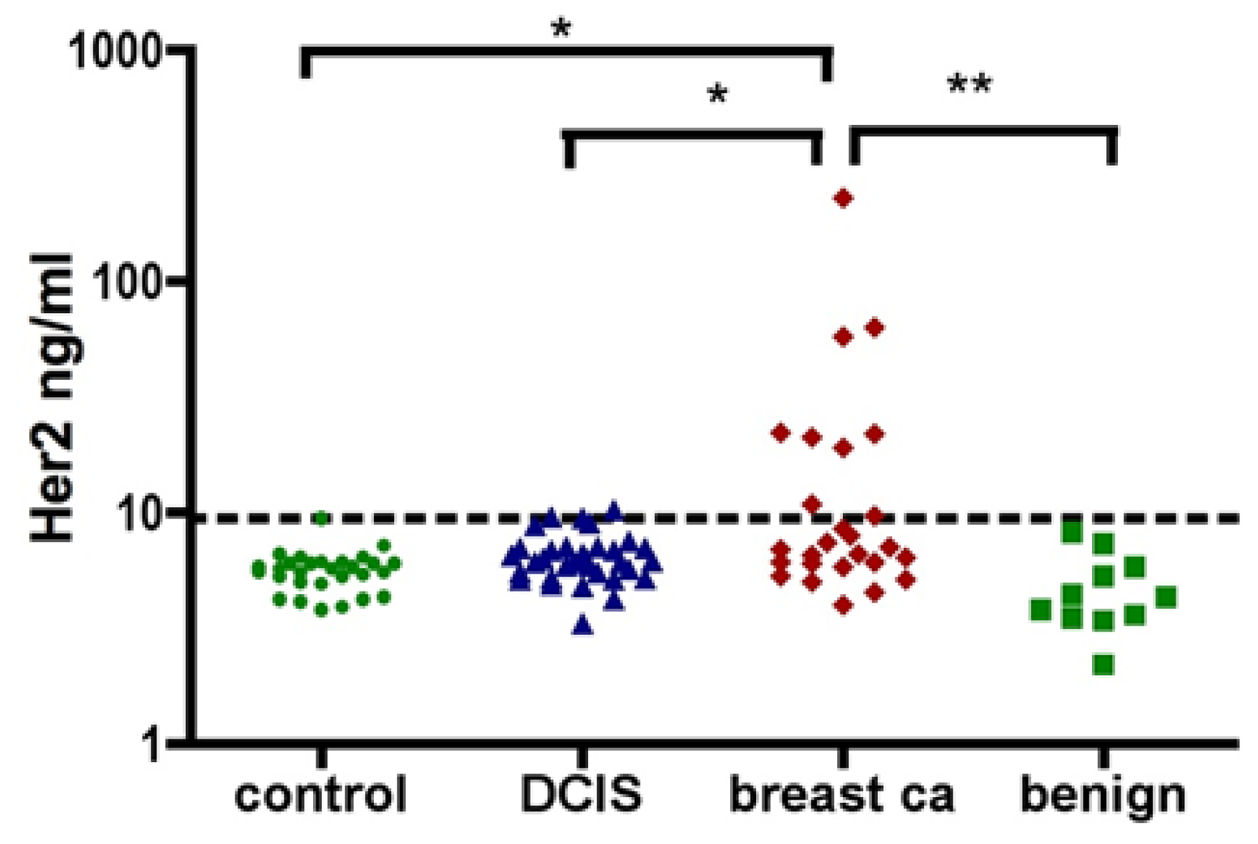
Serum HER2 ECD as determined by MBB-ELISA. The dotted line indicates the threshold level: 9.48 ng/ml. Samples were determined to have abnormal levels if their HER2 ECD concentrations were 4.

Serum samples included in the “breast cancer” group were collected from invasive breast cancer patients (stages II–IV, n = 28) who were diagnosed through the oncology clinics at the University of Pennsylvania and the MD Anderson Cancer Center. These patients received standard care for their diseases, which included chemotherapies and also targeted therapies for HER2 positive patients. The “DCIS” group (Figure 1) included serum samples from patients who had biopsy-proven DCIS. Control participants were healthy volunteers. The “benign” group referred to serum samples from patients with noncancerous breast diseases, including hyperplasia, cysts, etc. All participants were recruited according to a protocol approved by the Institutional Review Board (IRB) and serum samples were de-identified for blinded serum assays.

Serum samples from DCIS patients who were enrolled in the HER-2 (Human Leukocyte Antigen (HLA) class I & II peptides) pulsed dendritic cell vaccine trials [14,15] were also tested and shown in (Figure 4). Those patients had histologically confirmed DCIS with HER-2/neu over-expression (>2+ intensity) in at least 10% of cells. One pre-vaccine serum sample from each patient was collected. Serum samples were also collected right after the 1-month vaccination procedure and periodically during follow-up for up to 3 years.

### MBB ELISA for HER2 ECD

Anti-HER2 monoclonal antibodies m4D5 (provided by Genentech) and biotinylated 6E2 [16] were used as the capture and detection antibody, respectively. HER2 ECD standard was originally purchased from Oncogene Sciences. For ELISA, the capture antibody (m4D5, 5 μg/ml, 50 μL) was coated in PBS to a 96-well plate for overnight incubation at 4°C. After wash with PBST, the plate was blocked with 5% BSA for 1 h at room temperature (22°C). HER2 ECD standard and serum samples were diluted in the MBB buffer [13] and incubated for 1-h at 22°C. Diluted biotinylated detection antibody (50 μL, 1 μg/mL, with MBB) was added to each well for a 1 hr-incubation at room temperature. Streptavidin-conjugated horseradish peroxidase (HRP) (R&D systems) was used as the secondary antibody to detect the antigen-antibody complex. The plate was washed three times with PBST (0.1% Tween 20 in PBS) in-between incubations. Following six washes with PBST to remove excess detection antibodies, 50 μL of Tetramethyl Benzidine (TMB) substrate (0.1 mg/mL, 0.05 M phosphate-citrate buffer, pH 5.0) was incubated in each well at 22°C. The reaction was stopped within 15–30 min with 50 μL of 2 M H_2_SO_4_, and the data was collected at 450 nm (absorbance filter) using the SpectraFluor reader (Tecan).

### Immunohistochemical staining of DCIS lesions

Formalin-fixed, paraffin-embedded tissue blocks were sectioned at 5 μm on plus slides (Fisher Scientific, Hanover Park, IL). Sections were heated for 1 h at 60°C to remove excess paraffin, cooled for 10 min, and subsequently deparaffinized and rehydrated in a series of xylenes and alcohols. Immunohistochemistry was done using the DAKO Autostainer (DAKO, Carpinteria, CA) for HercepTest (DAKO) [14].

### Complement-dependent cytotoxicity (CDC) assay

CDC assay was performed on the HER-2/neu over-expressing breast cancer cell line SK-BR-3 as described before [14]. Briefly, 1 × 10^4^ cells were plated in quadruplets and incubated overnight at 37°C. 50 μL of human serum, which was inactivated at 56°C for 30 min and diluted 1:2 in PBS, was added to the cell cultures for 1 h. 20 μL of guinea pig complement (diluted 1:4; Sigma Chemical) was added to half of the wells. The other half served as antibody control. After 4 h, 15 μLWST1 was added to the wells. Plates were analyzed by an ELISA reader at the wavelength of 450. The percentage cytotoxicity was calculated using the following formula: [(a - b) / (a - c)] × 100(where a = cells in antibody only; b = cells in antibody plus complement; and c = medium only).

### Statistical tests

Statistical difference between groups was analyzed by the Student t test or by the non-parametric Kruskal-Wallis one-way analysis for multiple groups followed by the Dunn’s test. Survival curves, starting from the time when serum samples were collected, were plotted using the GraphPad Prism (version 4) software (San Diego, CA). This program calculates survival fractions using the Kaplan-Meier method to estimate survival as a function of time, and analyzes survival differences using the log-rank test.

## Result

### Serum HER2 ECD levels in breast cancer patients

With the help of the MBB buffer, we determined the HER2 ECD levels in serum samples from invasive breast cancer patients (“breast ca”, N=28), non-invasive DCIS patients (“DCIS”, N=33), patients with benign breast disease (“benign”, N=11), as well as healthy controls (“control”, N=29) (Figure 1). By the non-parametric Kruskal-Wallis one-way test, the serum HER2 ECD levels among these four groups were very significantly different (P=0.006). HER2 ECD levels in the invasive breast cancer group were very significantly different from those in the benign group (P<0.01, Dunn’s Multiple comparison test) and also significantly from those in the control group (P<0.05). The difference in the HER2 ECD levels between the DCIS group and the benign group was also significant (P<0.05).

The average HER2 ECD level for both the control and the benign group was 5.35 ng/ml (± 1.38, SD). The threshold level for abnormal levels was determined using this average plus 3× SD: 9.48 ng/ml. Within the breast cancer group, 9 out 28 samples (32%) had significantly elevated HER2 ECD levels. For DCIS, 2 out of 33 samples were positive (6%).

### Elevated HER2 ECD levels correlate with lower overall survival

**Figure 2:**
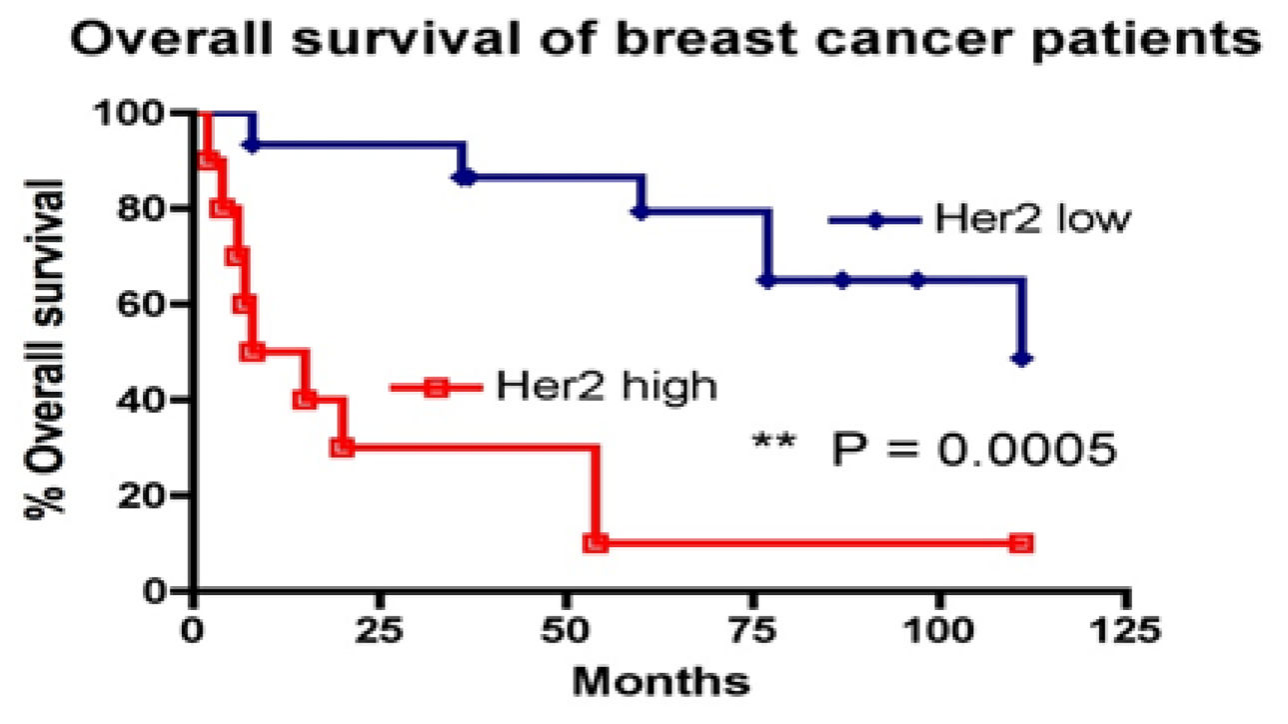
Serum HER2 ECD levels and overall survival in breast cancer. Kaplan–Meier survival curve of patients with high and low serum HER2 ECD levels. Months indicate the time after serum sample collections. Patients were under standard care and might have been exposed to clinical treatments at the time of serum collections.

As reported before, elevated serum HER2 ECD levels were reversely associated with overall survival [17]. For invasive breast cancer patients in our study, 25 had survival information. These patients might have received treatments previously but were divided into two groups to compare their overall survival under current cares: high serum HER2 ECD (>9.48 ng/ml) and low Her2 ECD groups (<9.48 ng/ml). Overall survival curves were calculated by the Kaplan-Meier method and compared by the log rank test (Figure 2). Patients with low HER2 ECD had significantly better survival (P=0.0005, logrank test).

### Changes of serum HER2 ECD levels in patients received HER2 vaccination

Previously, we have reported a vaccination trial in DCIS patients using HER-2/neu peptide pulsed dendritic cells [14]. Serum samples from 12 patients were collected before and periodically after vaccination for up to 30 months.

**Figure 3:**
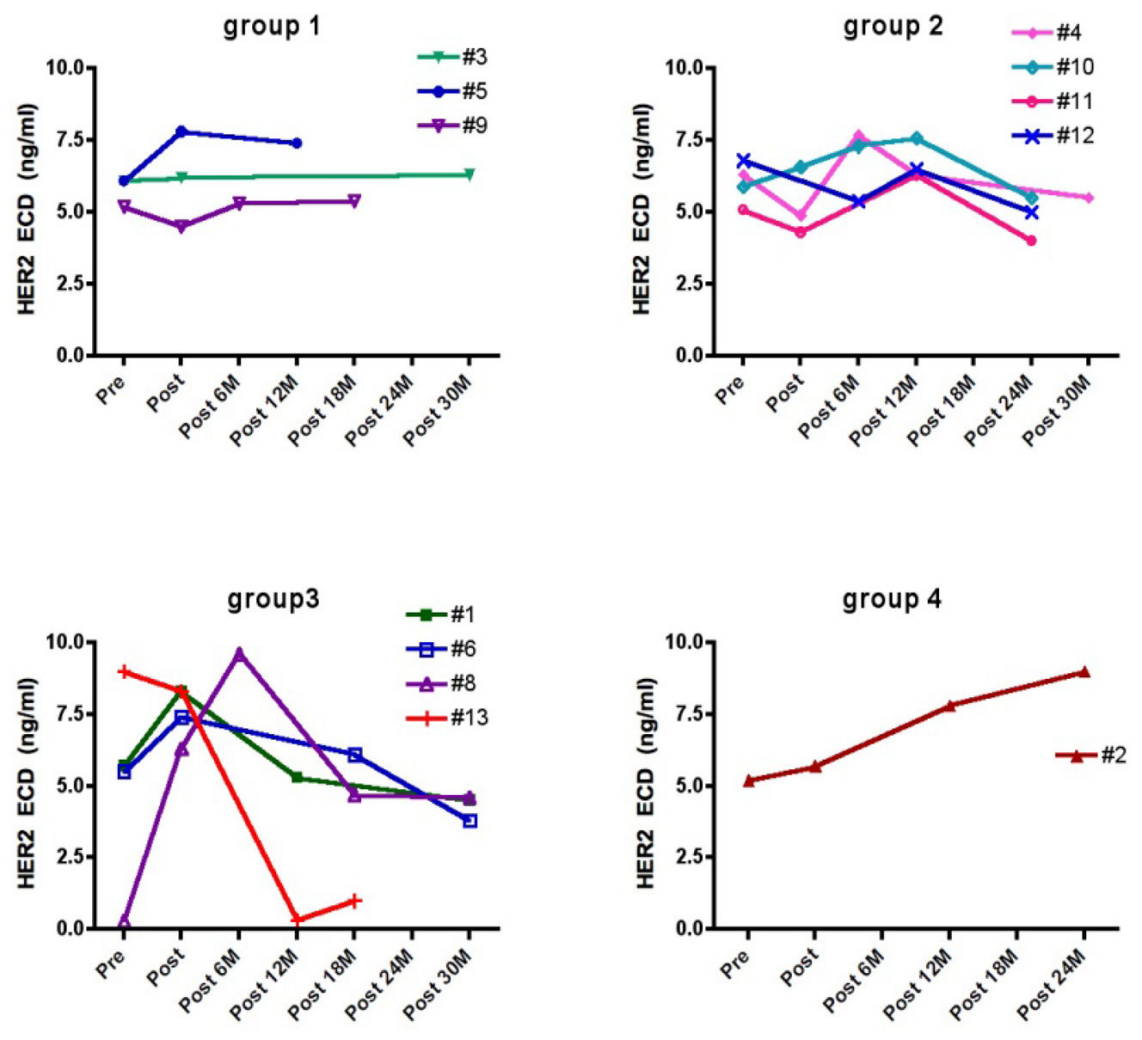
Time course of serum HER2 ECD in DCIS patients who received HER2/neu peptide vaccination. Serum samples from 12 subjects were collected before and after vaccination. All samples were tested in duplicate and the average was presented.

**Figure 4:**
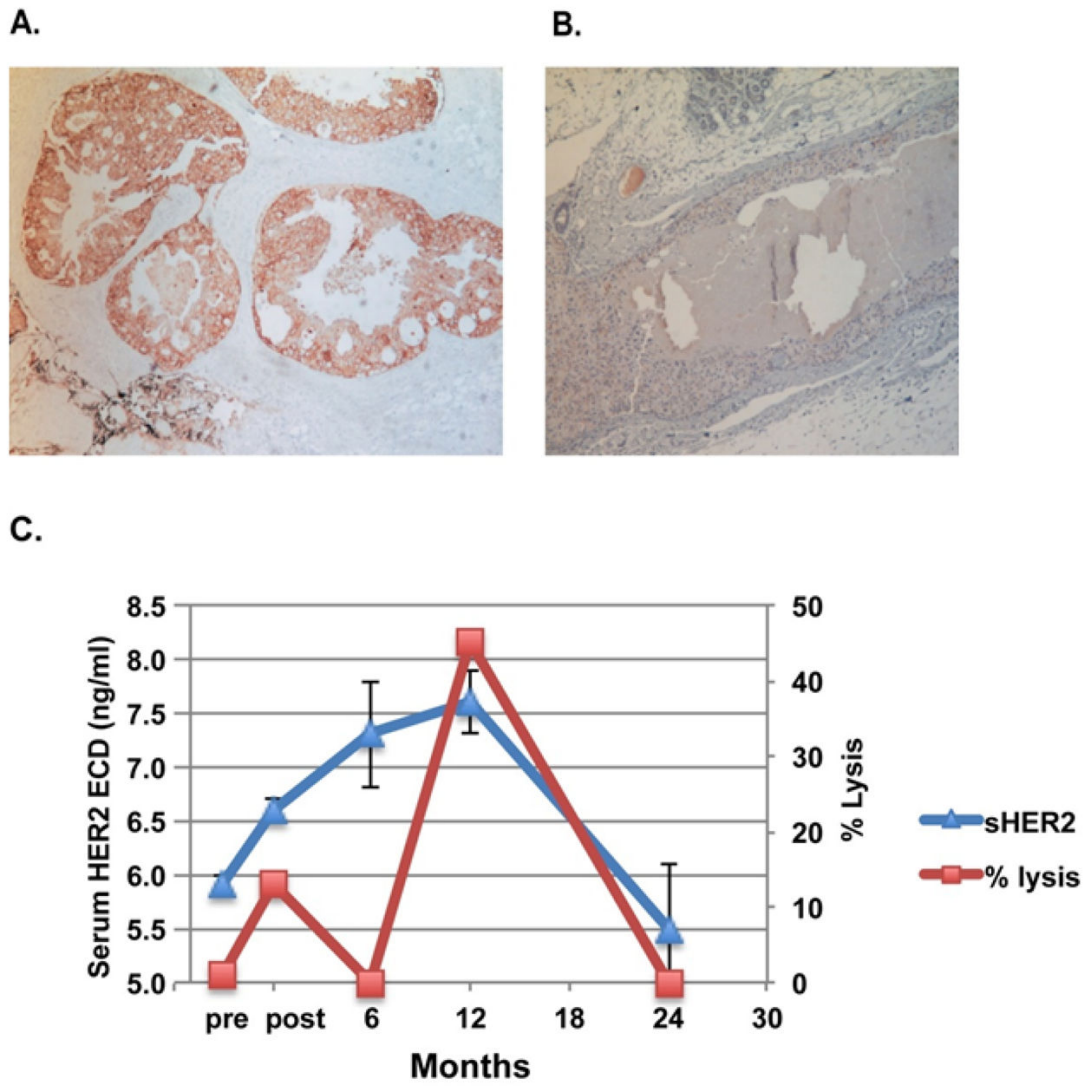
A case of recurrence after vaccination with phenotypic change in HER-2/neu expression. Immunohistochemical staining for tissue HER-2/neu (HercepTest). While the primary DCIS was HER2/neu positive, the emerged “recurred” tumor after vaccination from patient #10 was HER2/neu negative.

We analyzed these serum samples for HER2 ECD levels using the MBB-ELISA. As shown in Figure 3, these 12 patients can be divided into 4 groups. In group 1, HER2 ECD levels remained stable over the course up to 30 months. In group 2, HER2 ECD levels fluctuated but remained in a tight range (4–7.7 ng/ml). Compared with the pre-vaccination levels, all patients in this group had lower levels at 24–30 months after vaccination. Group 3 patients had larger change in serum HER2 ECD levels, with rising levels initially in 3 patients (e.g. #1, #6 and #8) but all patients in this group had much lower levels at later time points (> 45% drop from the highs). #13 showed a dramatic drop at post 12M, possibly as a result of chemotherapies received by this patient. Group 4 had only one patient (#2), whose HER2 ECD levels constantly went up (> 70% higher from the pre-vaccination baseline).

In follow-up, 2 out of these 12 patients had recurrence after the vaccination and surgery. Patient #2, the only one patient in Group 4 who had constantly rising HER2 ECD levels post vaccination, had relapse at year 5.

#10 also had recurrence but the tumor was determined as ER+/HER2− (Figure 4). We monitored the vaccination-induced anti-HER2 antibody activityin patient serum by examining complement dependent cytolysis activity against HER2/neu positive tumor cells [14]. The initial rising serum HER2 after vaccination (up to 12 months) was in parallel with the rising of anti-HER2 antibody activity, which peaked at 12 months post vaccination (Figure 4C), indicating the tumor cells under cytolysis due to vaccination could increase soluble HER2 in circulation. Apparently, the vaccination/surgery wiped out the original HER2 positive DCIS, and a different type of tumor emerged (ER+/HER2−) by escaping the vaccine-induced immunity and led to “recurred” tumor.

## Discussion

We have developed the MBB buffer to eliminate serum interference in sandwich ELISA assay [13]. Some serum samples contain large amount HAIA/HAMA and can introduce false positive signals in ELISA. As we have shown before, the MBB buffer was able to reduce these signals [9,13]. Previously, we used the MBB buffer to help detect several targets including IL8, Tyrosinase, and Cathepsin B from human serum samples. Here, we use this buffer for the detection of HER2 ECD.

As we observed in this study, 32% of the breast cancer patients had elevated levels of HER2 ECD in their serum. This serum positivity rate for HER2 ECD is very close to the average rate of 33% that was observed in breast cancer patients according to a survey of 70 studies [9].

The average rate represents a consensus on the existence of HER2 ECD in breast cancer. The rate is lower in primary (18%) but much higher in metastatic breast cancer (44%) [9]. As suggested by our study, serum interference of HAMA or HAIA will have limited influence on the population-based positivity rate. However, it will be a problem when an individual patient shows a false positive. A potential clue for possible false positive is the existence of unexpected high percentage of elevated HER2 levels in healthy control groups (4% – 22.2%) [18–21]. In this study, all samples in the control and benign breast disease groups had normal HER2 ECD levels by MBB-ELISA.

Serum HER2 ECD has been explored as a prognosis biomarker for survival. We confirmed in our study that the elevated HER2 ECD level was associated with reduced survival benefit in invasive breast cancer patients. We also further examined this in stage IV patients. Although a similar trend was observed, the difference was not statistical significant due to insufficient number of subjects (data not shown). We understood that patients in our study were not stratified for treatments received, which could raise concerns. However, we don’t think it is likely that the difference we observed in our study was affected by treatments. As these patients were under standard care, HER2 positive patients were routinely treated with trastuzumab based targeted therapies. If we could expect some influence from the therapeutic intervention, it would be an increase in the survival of HER2^+^ patients [22]. This would make it difficult for us to detect the difference in the survival between the two groups in (Figure 2). Nevertheless, we clearly observed significantly worse survival in patients with elevated serum levels of HER2. It is not clear if elevated serum HER2 level is associated with resistance to targeted therapies. We plan to follow up this study in a larger population of patients with well-controlled disease stages and treatments.

Our observation in the DCIS vaccination trial points to a clinical usefulness of serum HER2 testing. The current clinical practice of HER2 test (IHC and FISH) is dependent on the availability of tumor tissues, which limits the use of those tests, especially for monitoring patients. Here, only one patient (patient #2) had a constant rising HER2 ECD levels in serum after vaccination/surgery, and this patient turned out to have a relapse. Relapse also occurred in another patient (#10), but the “recurred” phenotype was HER2^−^. As the tumor cells of the HER2^+^ phenotype was wiped out by vaccine-induced cytotoxicity, it was not surprised to observe the drop of serum HER2 ECD levels.

Elimination of HER2^+^ tumor cells in DCIS patients by HER2-pulsed dendritic cell vaccine has been reported previously [15]. Out of 27 HER2^+^ patients(according to pre-vaccination biopsy), 5 patients had no trace of DCIS at post-vaccination surgical resection and another 11 patients showed complete loss of HER2 expression in residual DCIS. The current study examined long-term outcomes after vaccination/surgery and phenotypic change was observed in one out of two relapsed patients. Discordance in HER-2 status between primary and metastatic breast cancer tissues has also been reported [23]. The phenotypic change may reflect a mechanism for cancer to escape therapeutic treatments and/or immune surveillance.

A serum test for HER2 levels will be helpful for monitoring the phenotypic progression of the disease, and simultaneous monitoring of circulating ER and additional biomarkers [24] will provide more complete surveillance for disease progression. As both HER2 and ER status are relevant to therapeutic options [25–27], profiling these biomarkers in serum will facilitate clinical management of breast cancer patients. We are expanding our tests to a larger pool of DCIS vaccine patients. Additionally, we are also studying the HER2 levels in other breast cancer patients and will determine if there is a change in HER2 levels over the course of disease progression and treatments.

In summary, we have demonstrated a serum HER2 ECD ELISA assay that is facilitated by MBB-buffer to reduce interference. The assay is able to detect elevated serum HER2 in 32% of tested breast cancer patients while avoiding any unexpected false readings in healthy volunteers or patients with benign disease. Our studies indicate that by optimizing the assay condition, we could advance and translate the biomarker discovery research into clinical practices.

## Abbreviations

CDC: Complement-Dependent Cytotoxicity
DCIS: Ductal Carcinoma In Situ
ECD: Extra-Cellular Domain
ELISA: Enzyme-Linked Immunosorbent Assay
ER: Estrogen Receptor
FISH: Fluorescence In Situ Hybridization
HAMA: Human Anti-Mouse Antibody
HER2: Human Epidermal Growth Factor Receptor 2
HAIA: Human Anti-Animal Immunoglobulin Antibody
HLA: Human Leukocyte Antigen
HRP: Horseradish Peroxidase
IHC: Immunohistochemistry
IRB: Institutional Review Board
TMB: Tetramethyl Benzidine
